# Companionship and Sharing Create Social Connections of an Online Community-Based Intervention for Patients with Cancer Receiving Outpatient Care: Pilot Study

**DOI:** 10.2196/64977

**Published:** 2025-08-07

**Authors:** Yi He, Ying Pang, Ying Liu, Zimeng Li, Yan Wang, Yening Zhang, Zhongge Su, Lili Song, Shuangzhi He, Bingmei Wang, Lili Tang

**Affiliations:** 1 Key Laboratory of Carcinogenesis and Translational Research (Ministry of Education/Beijing), Department of Psycho-Oncology, Peking University Cancer Hospital & Institute, 52 Fucheng Road, Haidian District, Beijing, 100142, China, 86 1088196019; 2 Jiuzhong Technology (Beijing) Co., Ltd, Beijing, China

**Keywords:** mHealth, virtual community, social connection, outpatient, cancer

## Abstract

**Background:**

Online communities, platforms that facilitate social connections, have gained attention in the medical field, particularly for their potential to support patients. However, there is currently no online community specifically designed for patients with cancer receiving outpatient care. This study introduces a customized online community aimed at providing companionship and sharing to enhance the quality of life (QOL) among these patients.

**Objective:**

The purpose of this study was to assess the feasibility and initial effectiveness of a newly developed online community app in improving the QOL of patients with cancer receiving outpatient care.

**Methods:**

This pilot intervention-only study involved patients with cancer participating in a 4-week online community intervention through a mobile app. Eligible patients were aged 18 years or older, diagnosed with cancer, with an Eastern Cooperative Oncology Group Performance Status score of ≤2. The feasibility of the intervention was evaluated by community task participation rate, community task completion rate, and community daily login rate. Patients completed a QOL questionnaire (European Organization for Research and Treatment of Cancer Quality of Life Questionnaire Core 30, QLQ-C30) at baseline (T0), week 2 (T1), and week 4 (T2). After the intervention, participants were free to answer 3 questions about their user experience.

**Results:**

Baseline assessments were conducted on 30 patients, with 25 patients assessed at T1 (83.3%) and 22 at T2 (73.3%). The 4-week average community daily login rate was 60.37% (18.11/30 on average), with community task participation and community task completion rates reaching 42.25% (12.68/30 on average) and 22.38% (6.7/30 on average), respectively. Notably, after the study ended, participants continued logging into the app and completing tasks. Patients who actively engaged in community activities demonstrated significant improvements in global health status (mean 11.04, SD 10.3 vs mean –6.56, SD 11.58; *P*=.004), emotional function (mean 17.7, SD 22.93 vs mean –2.89, SD 13.9; *P*=.04), and constipation (mean 11, SD 16.5 vs mean 14.67, SD 17.39; *P*=.005) at T2, compared to those less active. The intervention enhanced emotional functioning and overall health and alleviated insomnia symptoms among active participants.

**Conclusions:**

The online community intervention, emphasizing companionship and sharing, was well accepted by patients with cancer and demonstrated initial effectiveness in enhancing the QOL. The study findings suggest that such interventions can provide a supportive environment for patients to cope with psychological, social, and physical challenges. Future validation of its effectiveness will require well-designed randomized controlled trials, and continued optimization tailored to specific user groups will be crucial to meet the evolving needs of the community. The core value of the online community lies in companionship and sharing, which can serve as a foundation for future research and development in this area.

## Introduction

Cancer and its treatments cause not only physical pain but also psychological, social, and spiritual suffering to patients. However, the lack of accessible psychosocial support resources, compounded by their uneven distribution, results in many patients with cancer being unable to receive the necessary rehabilitation guidance and psychological care [1,2]. Furthermore, disparities in cancer incidence and outcomes, influenced by socioeconomic status, race, and geographic location, exacerbate these challenges [3]. These inequalities underscore the urgent need to address gaps in health care and psychosocial support to improve patient outcomes.

The socioecological model [4,5] emphasizes the interaction between individual, interpersonal, community, and societal factors, with each level influencing causal relationships and preventive strategies. Despite this, existing interventions often focus predominantly on individual or interpersonal levels, with insufficient attention to community or societal dimensions. Social connections are considered a biological necessity directly linked to survival [4,5]. Patients with cancer often experience a lack of such connections, leading to social isolation and loneliness [6,7]. Recent studies have demonstrated that loneliness among cancer survivors is correlated with an increased risk of mortality [8]. Survivors who report feelings of loneliness face a significantly higher risk of death compared to those reporting low or no loneliness, with a clear dose–response relationship [8,9].

In this context, isolation refers to the sense of being disconnected from others and lacking companionship in real-world communities [10,11]. Community-level care can mitigate these effects by offering a platform for individuals to connect, build relationships, and share experiences and feelings [12]. It is important to note, however, that some individuals may still experience loneliness even within social circles [13]. The role of the community is thus to provide a safe environment where individuals can express inner worries and emotions [14].

As a result, extending psycho-oncology services to the community has become an essential component of care of patients with cancer. A recent intervention study led by community workers demonstrated that programs targeting low-income and minority patients with cancer significantly improved their quality of life (QOL) [15]. Nonetheless, several studies have identified barriers to the effective implementation of community care [16]. Unmet needs such as psychological adjustment, nutritional support, exercise management, and interpersonal communication are increasing among patients with cancer. These needs are frequently unmet at the community level, contributing to psychological distress and a diminished QOL for many patients.

Shifting from traditional real-world communities to online communities may offer an effective solution to overcoming these barriers [17-20]. Research indicates that one of the most promising aspects of eHealth is the growth of electronic communities, which create or enhance online communities based on shared interests to facilitate experience sharing and emotional support [17]. Online communities, facilitated through electronic media, have emerged as a viable intervention for mental health and social support [21]. For example, “PatientsLikeMe” is an online community that offers communication and support for individuals with various diseases [22]. On this platform, patients can connect with others who share similar experiences, fostering mutual understanding and emotional support [23]. These findings suggest that online care and community functions can substantially improve the QOL for patients with cancer.

In line with the socioecological model, our team has developed an online community for patients with cancer outside of the hospital setting. This community aims to provide a range of programs focused on companionship and mutual sharing, fostering social connections among patients and ultimately enhancing their QOL. The objective of this study is to assess the feasibility of implementing this intervention and to conduct an initial evaluation of its effectiveness in improving the QOL for patients with cancer.

## Methods

### Participants and Procedure

This pilot study adhered to the standards outlined in the Transparent Reporting of Evaluations with Nonrandomized Designs (TREND) guidelines [24]. Eligibility criteria were as follows: (1) age 18 years or older, (2) a confirmed cancer diagnosis via pathology, (3) an Eastern Cooperative Oncology Group Performance Status score of ≤2, and (4) fluency in Chinese with the ability to understand instructions provided during the online community process. Exclusion criteria were (1) inability to use a smartphone to participate in the online community, (2) a history of severe mental disorders, and (3) poor physical condition.

### Procedure and Data Collection

The study was conducted at Peking University Cancer Hospital & Institute, targeting patients with cancer receiving outpatient care. Recruitment occurred through direct outreach in outpatient clinics, referrals from collaborating oncologists, and online advertising via social media and cancer-related forums to identify potential participants. Upon obtaining informed consent, eligible patients had their characteristics and baseline assessment data collected. Participants then downloaded and engaged with an online community app under the guidance of the research team. Follow-up assessments were scheduled according to the study protocol.

The online community app automatically administered questionnaires at designated evaluation points, with assessments occurring at week 2 (T1) and week 4 (T2). After the 4-week intervention, the app remained accessible, allowing participants to continue using it voluntarily at their own discretion.

### Intervention

**Figure 1.**
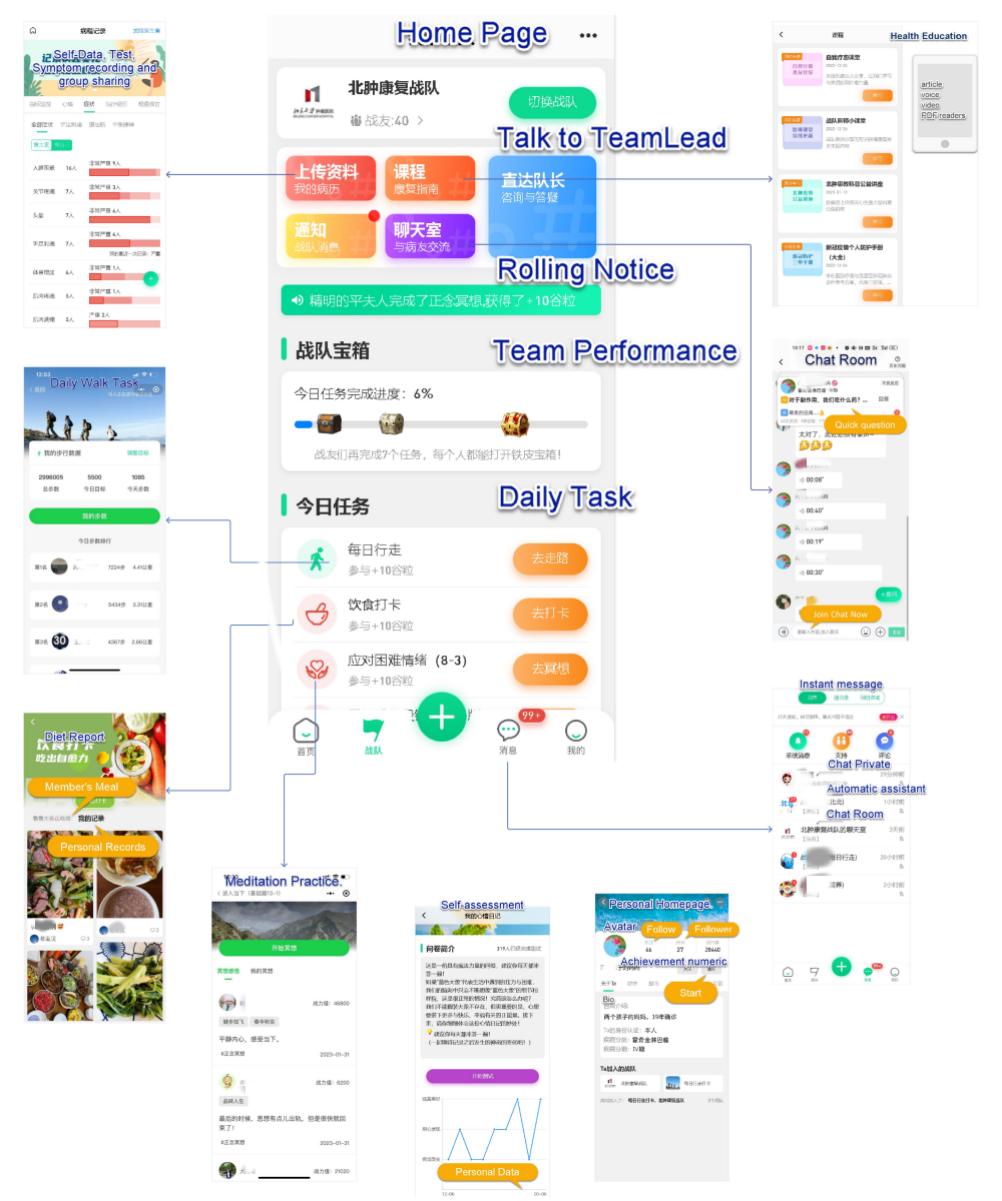
The specific intervention measures of the online community.

We developed a customized online community app named “Qianliu Health” (meaning “overcoming diseases together with thousands of companions, actively recovering” in Chinese) (Figures1  2). This app facilitated social connections for patients with cancer receiving outpatient care through features promoting companionship and sharing. Community activities included daily check-ins and patient-selected activities. Daily check-ins involved fixed-time notifications sent to participants, reminding them to engage in community activities, with recognition provided for completion. Patient-selected activities enabled participants to autonomously choose from a variety of community activities that matched their interests.

**Figure 2.**
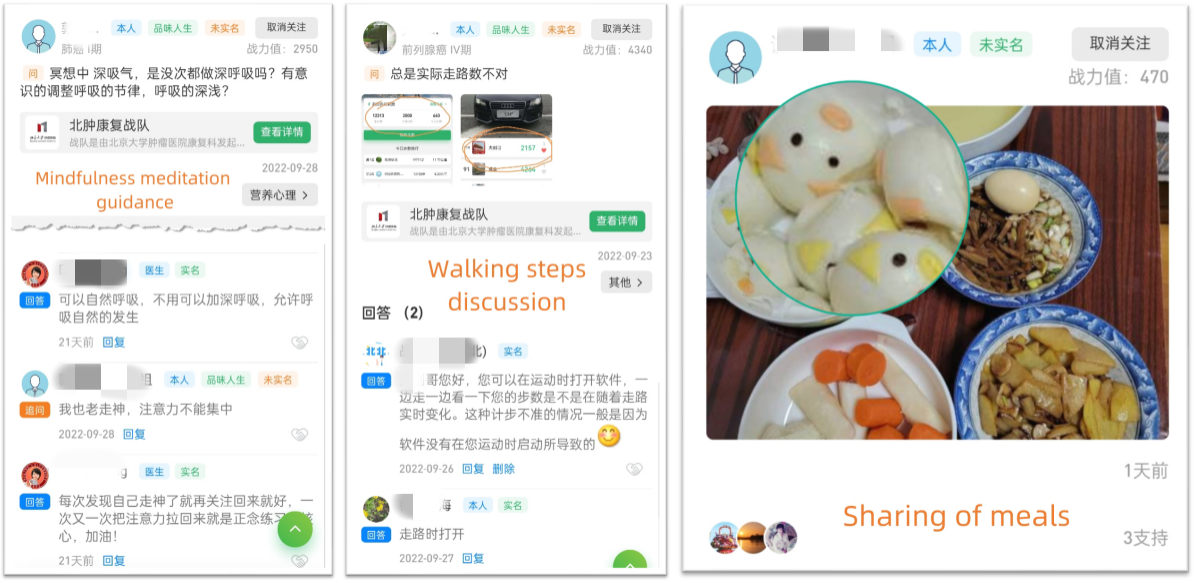
Screenshots of the app.

Sharing refers to the process through which participants exchange information and experiences, express emotions, and share important moments from their daily lives [25]. Specific tasks within the sharing component included: (1) users sharing daily meals for peer feedback and nutrition plan discussions (check-in task with daily platform reminders); (2) daily exercise check-ins, including step tracking and ranking within the community walking challenge (patient self-report and check-in task with daily platform reminders); (3) sharing personal achievements or daily highlights (non–check-in task and patient’s autonomous choice); and (4) interactive sharing within the community on topics such as disease, treatment, nutrition, stress, and symptom management (non–check-in task and patient’s autonomous choice).

Companionship refers to the provision of emotional support, social interaction, and a sense of belonging through the online community [26]. This component fosters a supportive environment where patients can connect with others facing similar challenges, share experiences, and offer mutual support. Specific tasks within the companionship component included (1) daily mindfulness meditation exercises, featuring 50 audio and video materials developed specifically for patients with cancer, allowing them to select preferred content for their practice, with regular supervision and support from professional therapists (check-in task with daily platform reminders); (2) setting up core buddies for direct one-on-one private chats, offering support and comfort, fostering companionship, and providing psychological support (noncheck-in task, patient’s autonomous choice); and (3) seeking assistance from other patients in chat rooms on topics such as disease, treatment, exercise, nutrition, stress, and symptom management (non–check-in task and patient’s autonomous choice).

To enhance participant engagement in community activities, the following strategies were implemented: (1) customized push notifications were sent 3 times daily (morning, noon, and evening) to encourage participation in designated community activities; (2) participants were awarded honors and personal growth points upon completion of each designated activity; (3) an automatic assistant was used to initiate discussions and promote active participation in chat rooms; (4) biweekly online community classrooms were hosted, where health care professionals conducted sharing sessions, fostering patient interaction within chat rooms; and (5) a team of 3 psycho-oncology-trained nurses contacted inactive participants by phone to encourage continued app usage.

### Measurement

#### Feasibility

Feasibility in this study was evaluated through several metrics. First, user engagement was assessed using the following metrics:

#### Community Task Participation Rate

This metric measures the proportion of participants who engage in at least one community activity (check-in task) per day.

Participants receive daily prompts to complete specific tasks, such as sharing meals or exercise check-ins. Engagement is recorded if participants complete at least one of these tasks on a given day. The participation rate is then calculated by dividing the number of days with at least one completed task by the total number of follow-up days.

#### Community Task Completion Rate

This metric evaluates the proportion of participants who complete all scheduled tasks (check-in tasks) per day.

Participants are prompted daily to complete multiple tasks, such as meal sharing, exercise check-ins, and meditation. Engagement is recorded if participants complete all scheduled tasks on a given day. The completion rate is then calculated by dividing the number of days with all tasks completed by the total number of follow-up days.

#### Community Daily Login Rate

This metric measures the proportion of days participants log into the app compared to the total number of follow-up days. Operationalization: The login rate is calculated by dividing the number of days participants log into the app by the total number of follow-up days. This metric serves as a general indicator of engagement with the online community.

#### Initial Effectiveness

Given the correlation between participation rate and the effectiveness of the online community intervention, participants were categorized based on their level of engagement into 2 groups: the active participants (4-wk daily login rate ≥70%) and inactive participants (4-wk daily login rate <70%). The initial effectiveness of the online community intervention was the change in the score on the QOL from baseline to 4 weeks between active or inactive participants, as measured by the European Organization for Research and Treatment of Cancer Quality of Life Questionnaire Core 30 (QLQ-C30), with a particular focus on the global health status and EF dimensions. The Chinese version of QLqQ-C30 consists of 30 items, with items 29 and 30 scored on a 7-point scale, while the remaining items are scored on a 4-point scale [27,28]. The instrument includes 15 domains: 5 functional domains (physical, role, cognitive, emotional, and social functioning), 3 symptom domains (fatigue, pain, and nausea or vomiting), 1 global health status domain, and 6 single items, each treated as a domain. These scores were measured and analyzed using standard scoring procedures in this study.

#### General Demographic and Disease Data Questionnaire

Demographic data collected included age, gender, education, and marital status. Disease-related information encompassed cancer diagnosis, disease stage, current cancer treatment status, current symptom treatment, and comorbidities.

#### User Survey

Following the intervention, participants were invited to respond to 3 open-ended questions regarding their user experience: (1) How was your experience in the online community? (2) Did you find the online community useful for you? Would you recommend it to others? (3) What do you think is the greatest value of the online community? These questions were delivered via in-app push notifications, and participants completed their responses directly within the app. No character limits were imposed on responses. All responses were collected and exported via the backend system. As this was not a formal qualitative study, no coding analysis was performed, and participant feedback was summarized inductively.

### Statistical Analysis

Based on previous similar studies [17,29-31], a convenience sample of 30 patients was selected to evaluate the feasibility and initial effectiveness of this study.

Baseline characteristics were summarized using mean and SD for continuous variables or number and percentage for categorical variables. Feasibility was assessed through the completion rate of community tasks. Differences between active and inactive participants in baseline characteristics and outcomes were assessed with the use of 2-sided Fisher exact tests and chi-square tests for categorical variables and independent-samples Student *t* tests for continuous variables. Assessments for QOL characteristics were conducted for active participants, and a paired *t* test was used to determine whether there was a statistically significant change between the baseline and week 4 scores.

SPSS software (version 17.0; IBM Corp) was used for data analysis. All *P* values were 2-sided, and *P*<.05 was considered statistically significant.

### Ethical Considerations

The study was reviewed and approved by the Institutional Review Board and Ethics Committee of Peking University Cancer Hospital (approval number 2022YJZ94) and was conducted between September 2022 and January 2023. All participants provided written informed consent prior to enrollment, ensuring they understood the study’s purpose, potential risks and benefits, and their rights as participants. To protect privacy and confidentiality, all data collected were anonymized or deidentified, with personal information securely stored and accessible only to authorized personnel. While no direct financial compensation was provided, participants received recognition and encouragement through community activities and personalized feedback, which were considered forms of nonmonetary compensation.

## Results

### Patient Characteristics

**Figure 3.**
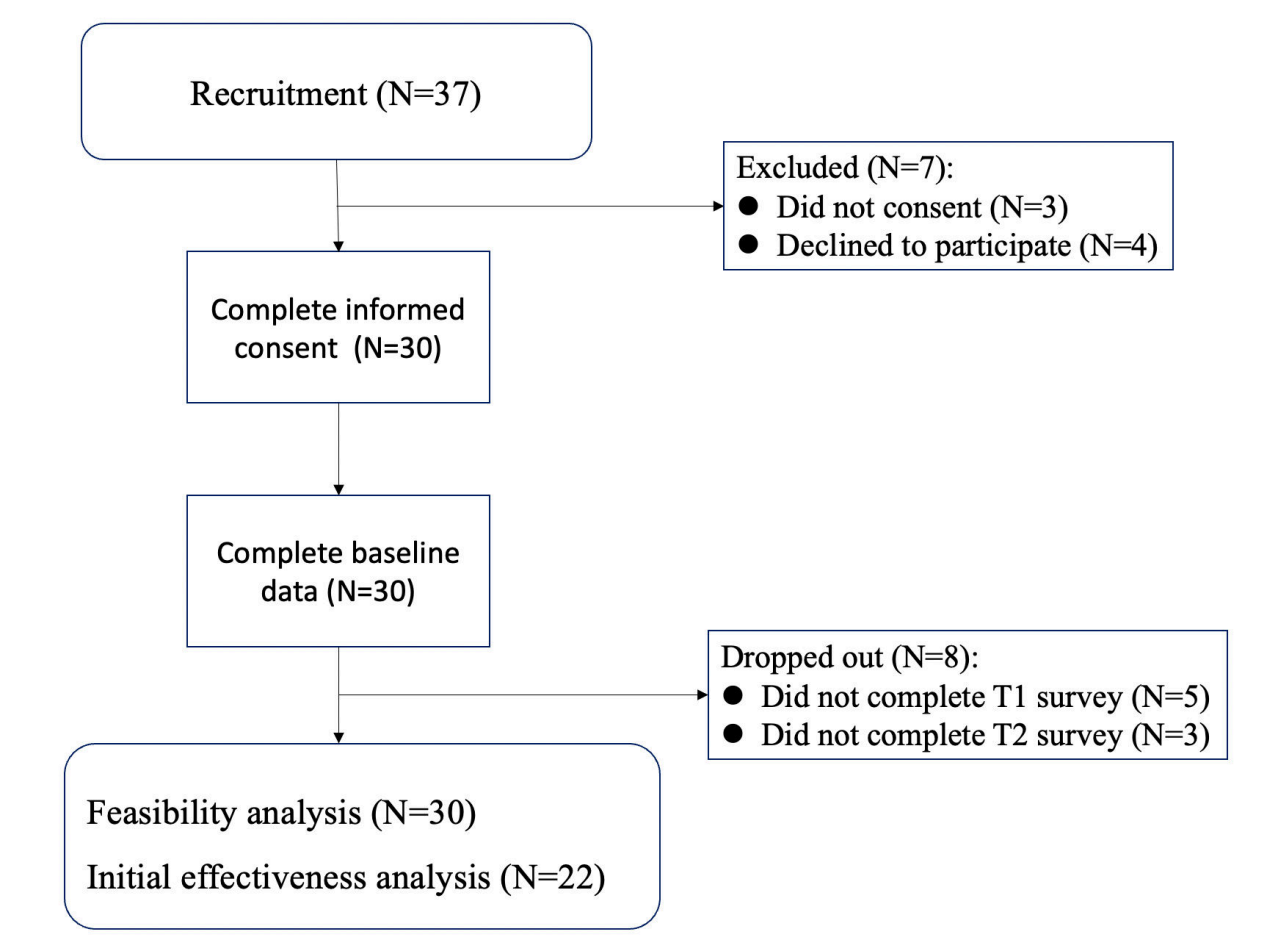
Study participant flowchart.

A total of 37 patients expressed interest in participating in the study, of which 30 patients were ultimately enrolled, yielding an overall participation rate of 81%. At the T1 assessment, 5 of 30 subjects (17%) were lost to follow-up, and at T2, 8 of 30 subjects (27%) were lost to follow-up, primarily due to worsening conditions and the need for hospitalization. Ultimately, 22 subjects were included in the analysis (Figure 3). Table 1 provides a summary of the baseline demographic and clinical characteristics of all 30 enrolled patients, while Table 2 presents the characteristics of the 22 participants included in the final analysis, categorized by their level of engagement (active or inactive).

**Table 1.** Baseline characteristics of the study participants (N=30).

Characteristics	Values
Age (years)	
Mean (SD)	53 (5)
Median (range)	54 (33‐74)
Marital status, n (%)	
Married	27 (90)
Single	3 (10)
Education, n (%)	
Middle school or high school	6 (20)
College or undergraduate	20 (66.7)
Master’s or doctorate	4 (13.3)
Sex, n (%)	
Male	10 (33.3)
Female	20 (66.7)
ECOG^a^ rating, n (%)	
0	10 (33.3)
1	14 (46.7)
2	6 (20)
Present stage of disease, n (%)	
No evidence of disease	1 (3.3)
Local recurrence	2 (6.7)
In situ or local	15 (50)
Metastasis	12 (40)
Current disease status, n (%)	
Complete response	5 (16.7)
Partial response	6 (20)
Stable disease	10 (33.3)
Progressive disease	6 (20)
Uncertain	3 (10)
Cancer treatment (past), n (%)	
Chemotherapy, immunotherapy, or endocrine therapy	19 (63.3)
Radiotherapy	8 (26.7)
Surgery	19 (63.3)
Cancer treatment (current), n (%)	
Chemotherapy, immunotherapy, or endocrine therapy	19 (63.3)
Radiotherapy	0 (0)
Surgery	1 (3.3)

a ECOG: Eastern Cooperative Oncology Group.

**Table 2.** The demographic characteristics of active and inactive participants.

Characteristics	Active participants (n=12)	Inactive participants (n=10)	*χ*^2^ (*df*)	*P* value^a^
Age (years), mean (SD)	51 (9)	58 (12)	−1.427 (1)	.17
ECOG PS^b^	0.7 (0.7)	0.9 (0.7)	−0.788 (1)	.44
Duration (months)	29 (74)	35 (53)	0.224 (1)	.83
Sex (male or female)	7/5	3/7	1.766 (1)	.23
Marriage (married or single)	10/2	10/0	1.833 (1)	.48
Prior surgery (yes or no)	7/5	8/2	1.180 (1)	.38
Prior radiotherapy (yes or no)	5/7	2/8	1.180 (1)	.38
Prior chemotherapy (yes or no)	6/6	7/3	0.903 (1)	.42
Metastatic (yes or no)	8/4	8/2	0.489 (1)	.65

a *P* values were calculated with the use of 2-sided *χ*2 and Fisher exact tests for categorical variables and the independent-samples Student *t* tests for continuous variables. *P*<.05 was considered statistically significant.

b ECOG PS: Eastern Cooperative Oncology Group Performance Status.

### Feasibility

The feasibility of the online community intervention was evaluated through several key metrics. The average daily login rate was 60.37% (18.11/30 on average) over the 4-week intervention period, with a range of 57.6% to 62.9%. The community task participation rate was 42.25% (12.68/30 on average), varying between 39.0% and 47.6%. The community task completion rate averaged 22.38% (6.7/30 on average), with a range of 19.6% to 25.3%. Poststudy engagement was also assessed, revealing that participants continued to interact with the app even after the intervention ended. A 15-week follow-up showed a daily login rate of 32.9% (an average of 4.28/13 participants), a task participation rate of 19.0% (an average of 2.47/13 participants), and a task completion rate of 6.6% (an average of 0.86/13 participants) (Table 3).

**Table 3.** The online community’s daily login rate, task participation rate, and task completion rate.

Stage	Week	Daily login rate (%)	Task participation rate (%)	Task completion rate (%)
Intervention	W1	62.9	42.9	25.3
Intervention	W2	57.6	39	20.1
Intervention	W3	62.9	47.6	24.5
Intervention	W4	58.1	39.5	19.6
Open-ended	W5	59.5	46.7	24.5
Open-ended	W6	46.2	36.7	18.6
Open-ended	W7	43.8	33.3	17.7
Open-ended	W8	38.1	32.4	16.1
Open-ended	W9	28.1	21.4	12.8
Open-ended	W10	30.5	20	12
Open-ended	W11	30	21.9	13.6
Open-ended	W12	32.4	25.2	14.7
Open-ended	W13	26.8	18.7	10.2
Open-ended	W14	23.6	14.2	4.8
Open-ended	W15	32.9	19	6.6

### Participation in the Online Community on Working Days Versus Nonworking Days

The average daily login rate on nonworking days (Saturday, Sunday, and holidays) was 55.6%, with 37.4% of members engaging in tasks (Multimedia Appendix 1). In contrast, on working days (Monday to Friday), the average daily login rate was 60.4%, with 44.7% of members participating in tasks. Regarding the number of rehabilitation actions (including daily meal logging or choosing different types of meditation) performed by participants, the average number of actions per individual on nonworking days was 8.8 (SD 2.9), compared to 7 (SD 3) on working days.

### The Initial Effectiveness of Online Community

Compared to inactive participants, active participants in the online community showed significantly greater improvements in the following QLQ-C30 dimensions: EF (mean 17.7, SD 22.93 vs mean −2.89, SD 13.9; *P*=.04), constipation (mean 11, SD 16.50 vs mean 14.67, SD 17.39; *P*=.005), and QL (mean 11.04, SD 10.3 vs mean −6.56, SD 11.58; *P*=.004) (Table 4).

**Table 4.** Mean (SD) change in QLQ-C30^a^ scores from baseline to 4 weeks between the active or inactive participant groups.

QLQ-C30 dimensions	Active participants (n=12), mean (SD)	Inactive participants (n=10), mean (SD)	*t* test (*df*)	*P* value^b^
Physical functioning	2.33 (11.16)	2.22 (18.41)	0.635 (20)	.54
Social functioning	9.30 (14.77)	1.89 (24.27)	1.181 (20)	.26
Emotional functioning	17.70 (22.93)	−2.89 (13.90)	2.304 (20)	.045
Cognitive functioning	1.89 (13.29)	0.11 (16.75)	0.249 (20)	.82
Role functioning	−3.78 (7.5)	18.44 (31.61)	1.354 (20)	.19
Dyspnea	11.00 (16.5)	7.33 (14.55)	0.500 (20)	.62
Appetite loss	11.07 (28.74)	3.67 (30.62)	–1.053 (20)	.31
Insomnia	25.85 (36.41)	−7.44 (27.77)	1.206 (20)	.26
Nausea and vomiting	5.59 (14.56)	5.33 (22.01)	−1.242 (20)	.23
Pain	1.89 (24.27)	1.78 (19.52)	−.011 (20)	.99
Fatigue	8.59 (17.26)	3.67 (14.55)	−1.629 (20)	.12
Constipation	11 (16.5)	14.67 (17.39)	3.212 (20)	.005
Diarrhea	−0.04 (16.58)	11 (16.50)	−1.415 (20)	.19
Financial difficulties	0.00 (16.5)	3.67 (11)	−0.555 (20)	.60
Global health	11.04 (10.3)	−6.56 (11.58)	3.406 (20)	.004

a QLQ-C30: Quality of Life Questionnaire Core 30.

b The *P* value was calculated with the use of 2-sided Student *t* tests for independent samples. *P*<.05 was considered statistically significant.

Intervention can enhance EF and overall health and improve insomnia symptoms among active patients. The EF (mean 63.54, SD 23.58 vs mean 87.63, SD 13.96; *P*=.03) and QL (mean 56.25, SD 13.33 vs mean 73.96, SD 11.23; *P*=.01) after intervention were significantly higher than before intervention for active participants. Insomnia (mean 49.96, SD 30.99 vs mean 20.67, SD 17.11; *P*=.04) after intervention was significantly lower than before intervention (Table 5).

**Table 5.** The intervention effects among active participants (n=12).

	Baseline, mean (SD)	4 weeks, mean (SD)	*t* test (*df*)	*P* value^a^
Physical functioning	82.46 (18.61)	84.17 (13.35)	−0.211 (11)	.85
Social functioning	77.17 (12.19)	87.46 (14.73)	−1.522 (11）	.15
Emotional functioning	63.54 (23.58)	87.63 (13.96)	−2.486 (11)	.04
Cognitive functioning	79.21 (14.63)	81.17 (13.84)	−0.275 (11)	.80
Role functioning	89.63 (15.15)	83.46 (17.68)	0.749 (11)	.48
Dyspnea	4.13 (111.67)	8.25 (15.28)	−0.607 (11)	.55
Appetite loss	16.58 (25.14)	4.13 (11.67)	1.272 (11)	.22
Insomnia	49.96 (30.99)	20.67 (17.11)	2.34 (11)	.04
Nausea and vomiting	8.42 (12.66)	2.13 (6.01)	1.27 (11)	.24
Pain	16.88 (21.93)	16.75 (17.77)	0.013 (11)	.99
Fatigue	38.92 (14.88)	24.78 (16.36)	1.808 (11)	.09
Constipation	12.50 (24.87)	20.75 (24.82)	−0.664 (11)	.53
Diarrhea	8.29 (15.35)	4.13 (11.67)	0.611 (11)	.55
Financial difficulties	8.25 (15.28)	12.54 (24.90)	−0.415 (11)	.68
Global health	56.25 (13.33)	73.96 (11.23)	−2.874 (11)	.01

a The *P* value was calculated with the use of a paired *t* test. *P*<.05 was considered statistically significant.

### User Survey

A total of 22 participants provided responses to the participation experience questions, among whom 14 explicitly stated that the online community was effective and that they would recommend it to others. Below, we present the experiences of 2 representative participants.

#### Case 1

A 67-year-old male patient with lung cancer, with a history of depression, initially expressed reluctance to participate, as he did not wish to focus on his illness. However, despite his verbal refusal, his daily login rate averaged 51%, and he completed 7 meditation tasks. Although he refrained from direct interaction and preferred to remain anonymous, his continued active participation in the community was a notable observation.

#### Case 2

A 54-year-old patient with breast cancer, currently undergoing hormonal therapy for 45 days, demonstrated a daily login rate of 100%. She consistently completed walking check-ins, dietary, and meditation tasks. While not highly proactive in initiating communication with others, she expressed a desire for feedback and engaged in private conversations with community members. Despite experiencing some anxiety, she reported that the community provided her with a stable and supportive environment for recovery.

## Discussion

### Principal Findings

This study evaluated the feasibility and initial effectiveness of an online community intervention designed to enhance QOL in patients with cancer receiving outpatient care. The findings suggest that such an intervention is feasible and may offer preliminary benefits. Specifically, participants with higher levels of engagement demonstrated greater improvements in selected QOL domains, including QL, EF, and CO, over a 4-week period compared to those with lower engagement.

### Comparison to Prior Work

Feasibility of the online community intervention was supported by acceptable task completion rates and consistent engagement patterns, comparable to those reported in similar eHealth and peer-support studies. Attrition, a well-documented challenge in digital health research, particularly in self-guided internet-based interventions, was relatively moderate in our study. Scholars have referred to this phenomenon as the “science of attrition” [32], and when compared to prior studies [33], our participation rate was relatively satisfactory. These findings suggest that the online community may have good clinical applicability.

As a pilot study, recruitment was intentionally broad rather than targeting a specific subgroup of patients. Interestingly, we observed that participants who engaged with the community on weekends and holidays demonstrated higher levels of activity and completed more community tasks. Moreover, a small subset of participants continued to engage with the platform beyond the study period, a behavior pattern previously described as “super user” dynamics [33]. These observations suggest that digital communities may particularly appeal to individuals who seek connection and shared experiences. However, future studies with larger samples are needed to better characterize these subgroups.

The user survey revealed diverse engagement patterns among patients. Some participants expressed initial reluctance but gradually found the community to be a safe and comfortable space for interaction. This aligns with previous research indicating that online cancer communities often facilitate information exchange, peer response, and resource sharing [34,35]. Passive engagement, such as reading rather than posting, was also observed, a pattern consistent with prior work identifying the role of observational participation in minimizing emotional risk [35]. Variability in engagement by disease stage has also been noted in studies of breast cancer communities, with more advanced-stage patients often engaging more deeply on an emotional level [36]. These findings highlight the importance of recognizing different participation styles and tailoring community features to accommodate diverse user needs.

In addition to feasibility, this study explored the initial effectiveness of the intervention. Notably, patients who were more active in the community experienced greater improvements in QOL scores from baseline to week 4, particularly in emotional functioning, global health status, and constipation symptoms. These exploratory findings align with previous research suggesting that social support and community engagement can alleviate emotional distress and enhance overall well-being among individuals with cancer [25,37]. Similar associations have been reported in studies of peer-support groups and online health communities [29,38].

The observed improvement in CO, while modest, may reflect the interaction between psychological and physical well-being, as has been suggested in prior research [39]. However, given the small sample size and nonrandomized design, these subgroup findings should be considered exploratory.

The finding that greater engagement may be associated with more favorable outcomes is consistent with research from larger digital platforms. For example, data from PatientsLikeMe indicate that active users report greater perceived benefits compared to less active users [22]. Encouraging meaningful engagement may therefore be an important consideration in the design and implementation of future community-based digital interventions.

### Limitations

This study has several limitations that must be considered when interpreting the findings. The small sample size, short intervention duration, and exploratory nature of subgroup analyses limit generalizability. The absence of randomization or stratified analysis increases the risk of bias and confounding. While the results are promising, they should not be interpreted as evidence of effectiveness. Rather, they underscore the potential value of further investigation.

### Conclusions

This pilot study demonstrated that an online community intervention for patients with cancer receiving outpatient care is feasible and well received. Exploratory findings suggest potential improvements in QOL among participants who actively engaged with the platform. Future research with larger samples and more rigorous designs is needed to evaluate effectiveness and inform implementation.

## Supplementary material

10.2196/64977 Multimedia Appendix 1 Participation in the online community on working days versus nonworking days.

## Abbreviations

CO: constipation
ECOG PS: Eastern Cooperative Oncology Group Performance Status
EF: emotional functioning
QL: global health status
QLQ-C30: Quality of Life Questionnaire Core 30
QOL: quality of life
TREND: Transparent Reporting of Evaluations with Nonrandomized Designs
