# Supplementary material for: Companionship and Sharing Create Social Connections of an Online Community-Based Intervention for Patients with Cancer Receiving Outpatient Care: Pilot Study
Source: JMIR Cancer. 2025 Aug 7;11:e64977. doi: 10.2196/64977 (PMC12331361; doi:10.2196/64977)
Supplement: Multimedia Appendix 1 [file cancer-v11-e64977-s001.docx]

**Table S1.**

| Day^a^ | Checking in for meals (n) | Mindfulness meditation (n) | Task participation rate (%) | Login (%) |
| --- | --- | --- | --- | --- |
| Monday | 47 | 66 | 41.40% | 57.00% |
| Tuesday | 54 | 64 | 47.10% | 58.80% |
| Wednesday | 54 | 77 | 42.70% | 58.00% |
| Thursday | 64 | 75 | 47.70% | 63.40% |
| Friday | 62 | 72 | 44.50% | 64.50% |
| Saturday | 58 | 79 | 36.20% | 55.70% |
| Sunday | 77 | 80 | 39.50% | 55.70% |
| Holiday | 44 | 54 | 36.70% | 55.30% |

^a^Table 4 only presents the data without any statistical analysis.
